# Potential applications of MEG3 in cancer diagnosis and prognosis

**DOI:** 10.18632/oncotarget.19931

**Published:** 2017-08-04

**Authors:** Yuqing He, Yanhong Luo, Biyu Liang, Lei Ye, Guangxing Lu, Weiming He

**Affiliations:** ^1^ Institute of Medical Systems Biology, Guangdong Medical University, Dongguan 523808, China; ^2^ Department of Epidemiology and Medical Statistics, Guangdong Medical University, Dongguan 523808, China; ^3^ Key Laboratory for Medical Molecular Diagnostics of Guangdong Province, Guangdong Medical University, Dongguan 523808, China

**Keywords:** cancer, long noncoding RNAs, maternally expressed gene 3, apoptosis, MEG3

## Abstract

LncRNAs are emerging as integral functional and regulatory components of normal biological activities and are now considered as critically involved in the development of different diseases including cancer. In this review, we summarized recent findings on maternally expressed gene 3 (MEG3), a noncoding lncRNA, locates in the imprinted DLK1–MEG3 locus on human chromosome 14q32.3 region. MEG3 is expressed in normal tissues but is either lost or decreased in many human tumors and tumor derived cell lines. Studies have demonstrated that MEG3 is associated with cancer initiation, progression, metastasis and chemo-resistance. MEG3 may affect the activities of TP53, MDM2, GDF15, RB1 and some other key cell cycle regulators. In addition, the level of MEG3 showed good correlation with cancer clinicopathological grade. In summary, MEGs is an RNA-based tumor suppressor and is involved in the etiology, progression, and chemosensitivity of cancers. The alteration of MEG3 levels in various cancers suggested the possibility of using MEG3 level for cancer diagnosis and prognosis.

## INTRODUCTION

Cancer is one of the main causes of death worldwide. A number of independent studies have demonstrated the involvement of noncoding RNAs, such as small nucleolar RNAs (snoRNAs), microRNAs (miRNAs), as well as long noncoding RNAs (lncRNAs) in cancer development [1–3]. LncRNAs are located in both intergenic and intronic regions of protein coding genes, and are frequently regulated and transcribed independently from the surrounding protein coding genes [4, 5]. Studies have shown that lncRNAs play an important role in transcriptional regulations by modulating promoter accessibility through chromatin reorganization by affecting processes like histone modification [6]. LncRNAs also affect the activity of miRNA, the subcellular localization of proteins and the production of endogenous siRNA [7–10].

Recent studies suggest some lncRNAs such as GAS5, p21, H19, HOTAIR and PTENP1 may play a role in tumor suppression by affecting cell proliferation, invasion and metastasis [11–16]. In addition, the expression of some lncRNAs is also associated with the effectiveness of cancer chemotherapy [2, 17, 18]. Therefore, some lncRNAs may be used as potential therapeutic targets for cancer treatment and biomarkers for the diagnosis and prognosis of cancers [19–23]. Maternally expressed gene 3 (MEG3) is an lncRNA which expresses in many normal tissues. However, it is frequently either lost, mutated or decreased level in many human tumors and tumor derived cell lines [22, 24, 25]. Restoring proper MEG3 expression level inhibits tumor cell proliferation and induces tumor cell apoptosis as well as autophagy [26, 27]. These findings suggest MEG3 is one of the lncRNAs with tumor suppressor activity [28].

In this review, we summarized recent studies on MEG3 associated aberrant expression and its effects in cancers, and explored the potential of using MEG3 as biomarker for cancer diagnosis and prognosis.

### The correlation of MEG3 expression with cancers

**Figure 1:**
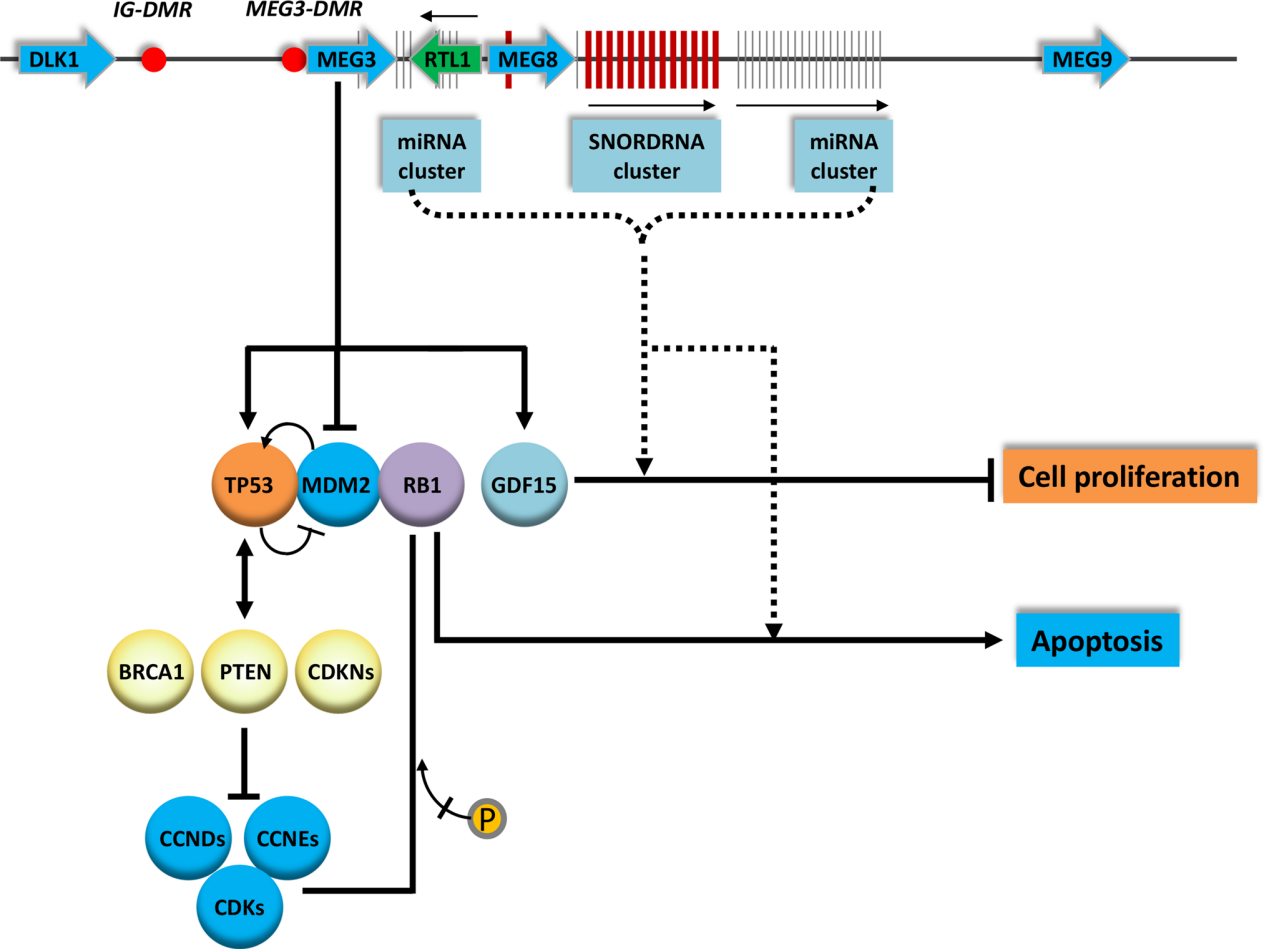
Genomic organization and schematic illustration on the involvement of MEG3 on cancer development The genes, mRNA and snoRNAs are indicated and the transcription orientations are indicated by arrows. The red dots represent differentially methylated regions. The schematic interactions of MEGs with key genes involved in cell proliferation and apoptosis are indicated. The arrows indicate activation. The doted lines are putative interactions.

MEG3 is located in the chromosome 14 DLK1–MEG3 imprinting region, containing multiple imprinted genes [25, 29, 30]. Besides MEG3, this region has a number of snoRNAs and miRNAs (Figure 1). Transcripts in this region are tightly regulated through methylation at the imprinting control regions [24, 25, 31]. The DLK1-MEG3 region has two key differentially methylated regions (DMRs) on the paternal allele, one called intergenic DMR (IG-DMR) located about 13kb upstream of the MEG3 transcription start site and the other, MEG3-DMR, overlapped with the MEG3 promoter region [32, 33]. The IG-DMR is the major imprinting control element and MEG3-DMR is responsible for the maintaining of proper allelic expression of transcripts in this region. The mature MEG3 RNA consists of ten exons with a length of about 1,600 nt [25]. Abundant levels of MEG3 have been shown in various tissues including brain, adrenal gland, placenta, testes, ovary, pancreas, spleen, mammary gland, and liver [25].

Recent studies demonstrated decreased MEG3 levels in a variety of primary human cancers (Supplementary Table 1) [16, 18, 24, 25, 31–73] and cancer derived cell lines (Table 1) [16, 18, 24, 31, 32, 34–36, 38, 41, 44, 46–54, 56, 57, 59, 60, 62–64, 74–77]. For example, MEG3 expression level is decreased in lung cancer [38], hepatocellular cancer [34], prostate cancer [37], multiple myeloma [33], meningioma [65], gastric cancer [35], and glioma [36]. In addition, loss of MEG3 expression was observed in the majority of nonfunctioning pituitary adenomas (NFPAs) [40, 45], neuroblastoma [31] and renal cell carcinoma [46]. Similar changes in MEG3 levels have also been found in various brain, bladder, bone marrow, breast, cervix, colon, liver, lung, meninges, and prostate cancers-derived cell lines (Table 1). Studies also reported that deletion of the MEG3 locus usually led to more aggressive cancers and MEG3 expression level correlated with tumor grade and prognosis in meningiomas [25], colorectal cancer (CRC) [54], NFPA [45], gastric cancer (GC) and cervical cancer [23, 49, 50]. For examples, Yin et al. analyzed 62 CRC cases and demonstrated that a lower MEG3 level correlates with lower pathological grade, deeper tumor invasion, and advanced TNM (tumor node metastasis) stage [54]. Li et al. found MEG3 expression level is significantly lower in invasive NFPAs compared to noninvasive NFPAs [45]. Sun et al. reported that down-regulated MEG3 is associated with poor prognosis and promotes cell proliferation in gastric cancer [50]. These findings suggest the level of MEG3 not only contributed to cancer development but also associated with pathological grade of the cancer and prognosis of the cancer patients. Recent meta-analysis studies also demonstrate the association of MEG3 level with cancer stages and survival outcome [22, 78].

**Table 1:** Changes of MEG3 expression level in different cancer cell lines

Reference	Cancer type	Cell lines	MEG3 Level	Methods	Potential function and mechanism
Li et al., 2017 [68]	AML	THP-1, HL-60, CCL-240 and CRL-1582	Down	qRT-PCR	MEG3 down regulates miR-184 expression in Leukemia
Lyu et al., 2017 [69]	AML	K562, TF-1, MOLM-13, U937, NB4, Kasumi-1, KG-1 and HL-60	Down	qRT-PCR	Dysregulation of MEG3 expression correlates with WT1 or TET2 mutations status, which probably plays an important role in AML pathogenesis.
Zhang et al., 2017 [71]	BC	MDA-MB-231, MCF-10A and MCF-7	Down	qRT-PCR	MEG3 expression associated with TNM stage and lymph nodes metastasis. Lower MEG3 predicted a poor DFS and OS for patients
Zhang et al., 2017 [23]	CC	HeLa and CaSki	Down	qRT-PCR	The low expression of MEG3 is likely due to promoter hypermethylation.
Hu et al., 2016 [74]	Pancreatic cancer	PANC-1 and SW1990 cells	Down	RT–PCR	Fenofibrate inhibits pancreatic cancer cells proliferation mediated by upregulation of MEG3
Li et al., 2016 [56]	Glioma	U251, U87 and A172	Down	qRT-PCR	DNMT1-mediated MEG3 hypermethylation causes the loss of MEG3 expression, followed by the inhibition of the p53 pathways
Zhang et al., 2016 [63]	CC	HeLa and CaSki	Down	qRT-PCR	MEG3 may interact with miR-21-5p to affects the post-transcriptional network
Zhou et al., 2015 [57]	GC	MKN45 and 7901	Down	qRT-PCR	miR-141 could interact with MEG3 and target E2F3
Peng et al., 2015 [59]	GC	HGC-27,MGC-803, MKN-45, SGC-7901, BGC-823 and AGS	Down	qRT-PCR	MEG3 could up-regulated Bcl-2 via its competing endogenous RNA activity on miR-181a
Luo et al., 2015 [60]	PC	PC3 and DU145	Down	qRT-PCR	MEG3 inhibited the expression of cell cycle regulatory protein Cyclin D1 and induced cell cycle arrest in G0/G1 phase
Yin et al., 2015 [54]	CRC	HCT-116 and DLD-1 cell lines	Down	qRT-PCR	MEG3 inhibits cell proliferation through TP53 activation.
Wang et al., 2015 [51]	RCC	RCC cell lines 786-0 and SN1	Down	qRT-PCR	MEG3 induces apoptosis by inhibiting the BCL-2 expression and activating the mitochondrial pathway.
Gao et al., 2015 [77]	RB	Retinoblastoma cell	Down	qRT-PCR	Decreased expression of MEG3 contributes to retinoblastoma progression and affects retinoblastoma cell growth by regulating the activity of Wnt/β-catenin pathway.
Zhuo et al., 2015 [34]	HCC	HCC cell lines	Down	qRT-PCR	UHRF1 regulates MEG3 level via DNMT1 and TP53
Xia et al., 2015 [18]	LC	Lung cancer cell lines A549/DDP	Down	qRT-PCR	MEG3 regulates TP53, β-catenin and survivin expression. Cisplatin treatment decreases the expression levels of MEG3.
Liu et al., 2015 [38]	LAD	A549/DDP cells	Down	Microarray	MEG3 induces the activation of TP53 and Bcl-xl in LAD cells.
Zhuang et al., 2015 [52]	MM	MSCs cells	Down	qRT-PCR	MEG3 played an essential role in osteogenic differentiation of the bone marrow MSCs, partly by activating BMP4 transcription.
Wang et al., 2015 [16]	PTC	TPC-1 and HTH83 cells	Down	qRT-PCR	RAC1 was negatively regulated by MEG3 at the post-transcriptional level, through a specific target site within the 3′UTR.
Modali et al., 2015 [62]	PNT	Mouse insulinoma cell lines	Down	qRT-PCR	DNA demethylating drugs reduce mouse insulinoma cell proliferation and restore MEG3 expression.
Parekh et al., 2015 [75]	MEN1	Menin-deficient fat-cell	Down	Microarray	Menin deficiency could result in fat cell hypertrophy and differential gene expression from the methylated MEG3 locus.
Qi et al., 2015 [76]	CRC	Colorectal cancer cell	Down	qRT-PCR	Decreased expression of MEG3 is associated with cell invasion and metastasis.
Yan et al., 2014 [35]	GC	SGC-7901 and BGC-823	Down	qRT-PCR	The suppression of miR-148a may contribute to the down-regulation of MEG3 in gastric cancer by modulating the activity of DNMT1.
Sun et al., 2014 [50]	GC	GC cell lines	Down	qRT-PCR	MEG3 may function as a tumor suppressor by activating TP53 in gastric cancer.
Sheng et al., 2014 [48]	EOC	Ovarian cancer cell lines	Down	qRT-PCR	Decreased expression of MEG3 in EOC is due to promoter hypermethylation.
Greife et al., 2014 [24]	UC	Urothelial cancer cell lines	Down	qRT-PCR	Down regulation of DLK1 and MEG3 is caused by DNA hypermethylation.
Jia et al., 2014 [47]	TSCC	SCC-15 and CAL27	Down	qRT-PCR	Antitumor effects of MEG3 are mediated by TP53 activation in TSCC.
Qin et al., 2013 [49]	CC	CC lines HeLa and C-33A	Down	qRT-PCR	MEG3 induces G2/M cell cycle arrest and apoptosis.
Lu et al., 2013 [44]	NSCLC	NSCLC cell lines	Down	qRT-PCR	Decreased MEG3 level in NSCLC tissues could be affected by DNA methylation. MEG3 regulates cell proliferation and apoptosis via activation of TP53.
Ying et al., 2013 [53]	BC	Bladder cancer cells	Down	qRT-PCR	Downregulated MEG3 activates autophagy and increases cell proliferation in BC.
Wang et al., 2012 [36]	Glioma	U251 and U87 MG cells	Down	qRT-PCR	MEG3 expression is decreased in glioma cell lines and effects TP53 and genes required for TP53 activation.
Braconi et al., 2011 [41]	HCC	HCC cell lines (PLC/ PRF/5)	Down	qRT-PCR	Deregulated miR-29a level in HCC affects MEG3 expression through promoter hypermethylation.
Kawakami et al., 2006 [46]	RCC	Human renal cells	Lost	RT–PCR	Gain of methylation upstream of MEG3 leads to down regulation of DLK1 in RCC.
Astuti et al., 2005 [31]	NB	Neuroblastoma cell	Lost	RT-PCR	The loss of MEG3 expression is associated with MEG3-DMR hypermethylation.
Zhao et al., 2005 [32]	PT	MCF7 and HeLa cells	Lost	qRT-PCR	Hypermethylation of the promotor region is associated with the loss of MEG3 expression.
Zhang et al., 2003 [64]	NFPA	HeLa, MCF-7, Neuroglioma H4	Lost	RT-PCR	MEG3 has a strong ability to inhibit proliferation of several carcinoma cell lines and NFPA.

**Abbreviations: CRC,** Colorectal cancer; **CC**, cervical cancer; **RCC**, Renal Cell Carcinoma; **RB**, Retino-blastoma; HCC, Hepatocellular Carcinoma; **LC**, lung cancer; **LAD**; lung adenocarcinoma; **MM**, Multiple Myeloma; **PTC**, papillary thyroid carcinoma; **MEN1**, multiple endocrine neoplasia type 1; **GC**, gastric cancer; **EOC**, epithelial ovarian cancer; **UC**, urothelial carcinoma; **TSCC**, tongue squamous cell carcinoma; **BC**, bladder cancer; **NSCLC**, Non-small cell lung cancer; **NB**, neuroblastoma; **PT**, Pituitary Tumors; **PNT**, pancreatic neuroendocrine tumor; **DNMT1**, DNA (cytosine-5-)-methyltransferase 1; **M-PCR**, Methylation-Specific Polymerase Chain Reaction; **DLK1**, delta-like 1 homolog; **UHRF1**, ubiquitin-like with PHD and ring finger domains 1; **MSCs**, Mesenchymal stromal cells.

### MEG3 inhibits cell proliferation and induces apoptosis in cancer

Restoring the expression of MEG3 impedes cancer cell proliferation *in vitro*. For example, MEG3 inhibits proliferation and induces apoptosis in cancer cell lines including MG63, OS-732, SaOS, G292, and 143B (osteosarcoma) [79], OVCAR3 and A2780 [26], MDA-MB-231, MCF-10A and MCF7 (breast) [27], HeLa (cervix), C-33A (cervix) [49], A549/DPP (lung) [38], PRC/PRF/5 (liver) [41], U251 (brain) U87MG (brain) [36], HCT116 (colon) and DLD1 (colon) [54] (Table 1). There is also ample evidence showing MEG3 may affect the growth and differentiation of cells *in vivo* (Table 2). For example, studies have shown that restoring the expression of *MEG3* suppresses tumor growth in nude mice (Table 2) [34, 38, 44, 46, 54, 60, 62, 80–82].

**Table 2:** Changes of MEG3 expression level in different mouse models

Reference	Cancer Type	Country	Samples	Number of samples case/control	MEG3 Level	Methods	Potential function and mechanism
Chunharojrith et al., 2015 [80]	NFAs	USA	Nude mice	5/5	Down	RT-PCR	MEG3 causes cell cycle arrest at the G1 phase
Luo et al., 2015 [60]	PC	China	Nude mice	5/5(pCDNA /pCDNA-MEG3)	Down	qRT-PCR	MEG3 inhibits the expression of cell cycle regulatory protein Cyclin D1 and induced cell cycle arrest in G0/G1 phase
Yin et al., 2015 [54]	CRC	China	Nude mice	3/3	Down	qRT-PCR	The proliferation index ki-67 is significantly decreased in the MEG3-transfected tumor cells. In addition, the cleaved caspase-3 level is increased.
Zhuo et al., 2015 [34]	HCC	China	Nude mice	5/5	Down	qRT-PCR	MEG3 inhibits proliferation and induces apoptosis through the accumulation of TP53.
Liu et al., 2015 [38]	LAD	China	Nude mouse xenograft model	6/6 (pCDNA-MEG3/ empty vector + cisplatin)	Down	qRT-PCR	MEG3 overexpression increases the *in vivo* chemosensitivity of LAD cells to cisplatin.
Modali et al., 2015 [62]	Insulinomas	USA	Mice	7/6	Down	qRT-PCR	DNA demethylating drugs reduce mouse insulinoma cell proliferation and restore MEG3 expression.
Lu et al., 2013 [44]	NSCLC	China	Nude mice	5/5	Down	qRT-PCR	Overexpression of MEG3 could inhibit tumor growth *in vivo*.
Lempiäinen et al., 2013 [82]	LT	UK	PB treatment Mice	5 Pairs	Down	qRT-PCR	PB induces MEG3 expression in glutamine synthetase positive hypertrophic hepatocytes.
Gordon et al., 2010 [81]	PA	USA	Nude mice	5 Pairs	Lost	qRT-PCR	Increased expression of angiogenic genes in the brains of MEG3-null mice.
Kawakami et al., 2006 [46]	RCC	Japan	Nude mice	5 Pairs	Lost	qRT-PCR	Reintroduction of DLK1 into DLK1-null RCC cell suppresses tumor growth in nude mice.

**Abbreviations: CRC**; Colorectal cancer; **HCC**, Hepatocellular Carcinoma; **LAD**; lung adenocarcinoma, **PA**, pituitary adenomas; **RCC**, Renal Cell Carcinoma; **LT**, Liver Tumor; **NSCLC**, Non-small cell lung cancer.; **NFAs**, non-functioning pituitary adenomas.

Several Meg3 knockout (KO) mouse models have been used to study the function of MEG3 *in vivo*. The KO mouse model created by Zhou et al. carries a deletion of a 5 kb genomic region containing the first five exons and a small portion of the Meg3 promoter [81]. Mice carrying the paternal allele deletion are alive and normal. However, mice with deletion at the maternal allele died perinatally with major skeletal muscle defects, and the expression of both Meg3 and the downstream Meg8 was not detectable. Takahashi et al. created another Meg3 KO mouse with a 10 kb deletion of the genome containing the MEG3-DMR and the first five exons of the Meg3 gene [83]; the mice with deletion on the maternal allele died 4 weeks after birth. Surprisingly, mice with homozygous deletion survived and grew to fertile adults [83]. In Takahashi's KO mice, it did not affect the methylation status of the IG-DMR [83], but in Zhou's KO mice the IG-DMR region is hypermethylated [81]. This suggests MEG3-DMR may affect the methylation status of IG-DMR and once Meg3-DMR is deleted the IG-DMR will be methylated. Therefore, one of the possible functions of Meg3-DMR is to maintain an active (unmethylated) status in the IG-DMR region which allows the expression of downstream MEGs. A Meg3 KO generated by Gordon et al. showed an increase of brain microvessel formation in the Meg3 null embryos. This finding suggests MEG3 may affect the activities of genes involved in VEGF angiogenic pathway [81, 84].

### Epigenetic regulation of transcripts in the MEG3 region

#### MEG3 and DNA methylation

Different mechanisms may contribute to the decrease or loss of MEG3 expression in cancers, including hypermethylation of the regulatory regions and deletions of the gene, as well as post translational degradation via miRNAs. Among them the hypermethylation in the MEG3 promoter and IG-DMR regions probably play the most important role for the decrease of MEG3 expression in cancers [32, 33]. The MEG3 promoter region is GC-rich and overlaps with the MEG3-DMR. The expression of MEG3 can be modulated by changing the methylation state of its promoter and MEG3-DMR regions [85, 86]. For example, in human NFAPs the methylation levels in both the IG-DMR and MEG3 promoter regions are higher than in normal pituitary [32, 40]. Astuti et al. also showed that the MEG3-DMR is completely methylated in neuroblastoma cell lines and hypermethylated MEG3 promoter is associated with down regulation of MEG3 and upregulation of DLK1 expression [31]. In neuroblastoma and pheochromocytoma tissues, the aberrant methylation of the MEG3 promoter correlated with decreased MEG3 level in 25% and 10% of the cases, respectively [31]. The silenced MEG3 expression in ovarian cancer is also due to promoter region hypermethylation which may also contribute to the progression of cancer development [33, 48]. Increased methylation in IG-DMR was also found in ovarian cancer and NSCLC (non-small cell lung cancer) derived cell lines [44, 48].

The degree of IG-DMR methylation showed a positive correlation with tumor grade and overall survival (OS) [25]. For example, the percentages of methylated CpG in IG-DMR are 50.1, 56.4, 61.0 and 68.8% in normal meninges, grade I, grade II, and grade III meningiomas respectively [25]. The level of MEG3 promoter region (MEG3-DMR) methylation is associated with OS of acute myeloid leukemia (AML) patients with myelodysplastic syndrome (MDS). The MEG3-DMR hypermethylation can be detected in 50% of patients with AML with MDS compared to only 34.9% of the patients with just MDS [85]. These studies suggest aberrant methylation of the MEG3 locus may be accentuated during cancer progression and the degree of MEG3 suppression is associated with the overall aggressiveness of cancers.

#### snoRNAs

SnoRNAs are involved in post-transcriptional modifications and maturation of ribosomal RNAs and transfer RNAs. It can be divided into two large families: C/D box containing snoRNAs and H/ACA box snoRNAs. The C/D box snoRNAs are mainly responsible for methylation (2′-O-methylation) and H/ACA snoRNAs are for pseudouridylation of ribosomal RNAs [87, 88]. The MEG3 genomic region contains a cluster of C/D box snoRNAs - member of the SNORD112 (1 copy), SNORD113 (9 copies) and SNORD114 (31 copies) families [89, 90] (Figure 1). The SNORD112 and some of the SNORD113 members are located in the intron of MEG8 gene. Like what has been observed in MEG3, some snoRNA expression levels were also decreased in AML and ALL cells compared to normal cells [91]. Opposite to the effect of MEG3, over-expressing SNORD114 member induces K562 and HCT116 cell proliferation [92] and suppressing the snoRNA induces cell death. Further studies revealed that SNORD114 promotes cell cycle progression through G0/G1 to S phase transition [91]. The level of SNORD114-3, one of the snoRNAs in the DLK1-MEG3 snoRNAs cluster showed good correlation with MEG3 expression, which suggests the SNORD114-3 and MEG3 probably are co-regulated by the IG-DMR and MEG3-DMR regions. These findings indicate some of the snoRNAs in the region may have similar involvement in cell proliferation and tumor progression as MEG3.

#### microRNAs

MiRNAs are involved in the regulation of all aspects of cellular function including cytokine signaling cascades, DNA methylation, oncogenic kinase expression, and others that are important for the development and progression of cancers [61, 93–95]. Besides snoRNAs, the MEG3 region contains a number of miRNAs and some of these are located in the intron of MEG3, MEG8 and RTL1 (retrotransposon-like 1) transcripts (Figure 1). Some miRNAs in the locus are probably derived from the MEG3 primary transcript and under MEG3 promoter control [96–98]. Like MEG3, the levels of these miRNAs have also been shown to affect cancer development and influence cancer cell chemosensitivity, which is directly linked to the prognostic outcome of different cancers. For example, Shih et al. reported 29 miRNAs that were associated with disease outcome in advanced ovarian cancer patients and 11 of the 29 miRNAs are located in the DLK1-MEG3 cluster [99]. Nine of those 11 miRNAs including miR-433, miR-127, miR-381, miR-377, miR-299-3p, miR-409-3p, miR-154, miR-382, and miR-376c are associated with OS. In a separate study, the increase of miR-376c level in ovarian cancer cells was found to inhibit cisplatin induced cell death [100]. The miRNAs in this region have also been shown to be involved in the progression of esophageal squamous cell carcinoma (ESCC) and hepatocellular carcinoma (HCC) [101–105]. In addition, miR-495, miR-134, miR-409-3p, miR-496, miR-379, miR-369-3p in the cluster are linked to the tumor invasion depth in gastric cancer [104, 106]. Besides that miR-376c has been shown to be associated with nodal metastasis in gastric cancer and miR-494 is significantly correlated with gastric cancer stage. The study also indicated that miR-495, miR-433, and miR-410 levels can be used to predict both disease free survival (DFS) and OS in gastric cancer patients [104]. The methylation pattern in the MEG3 DMR as well as the expression profile of miRNAs in the region can distinguish high-aggressiveness versus low-aggressiveness osteosarcoma cell lines, and the levels of miR-495, miR-329, miR-487b, miR-410, and miR-656 can predict the outcome of patients with osteosarcoma [107]. In addition, the expression pattern of miRNAs in the MEG3 region can also identify previously unrecognized distinct molecular subtypes of osteosarcoma. These findings suggest the possible therapeutic implications of miRNAs in the region.

LncRNAs play crucial roles in epigenetic regulation of gene expression through interactions with miRNAs, mRNAs and proteins [108]. MiRNAs can directly or indirectly affect the lncRNA expression level. Therefore, besides the changes of protein coding genes, it is necessary to investigate the epigenetic regulation of noncoding RNAs and the inter-relationship between miRNAs and lncRNAs to understand the underlying molecular processes involved in cancer development and progression. In a recent study, the down-regulated MEG3 in cervical cancer has been shown to affect cell proliferation and apoptosis through modulating the level of miR-21-5p [63]. In another study, the levels of miR-141 and MEG3 have been found to be significantly reduced in GC patients. Furthermore, E2F3 was identified as a target of miR-141, and its expression level was also found to be negatively associated with both MEG3 and miR-141 [57]. These findings may indicate the interaction between miR-141 and MEG3 to inhibit GC cell proliferation. Studies also showed that miR-29 may involve in the regulation of MEG3 level which correlates with a poor prognosis of HCC [41, 109]. These data suggest the involvement of miRNAs on tumor progression may be in part mediated through MEG3 activity.

Loss of imprinting/methylation changes in the 14q32 non-coding region defines reproducible previously unrecognized osteosarcoma subtypes with distinct transcriptional programs and biologic and clinical behavior. Future studies will define the precise relationship between 14q32 imprinting, non-coding RNA expression, genomic enhancer binding, and tumor aggressiveness, with possible therapeutic implications for both early- and advanced-stage patients.

### MEG3 involved in key cancer associated signaling pathways

#### p53 pathway

The *p53* (*TP53*) gene encodes a transcription factor, TP53, which has been associated with tumor development and growth [110]. The activation of TP53 leads to cell cycle arrest, replicative senescence, and/or apoptosis [111]. TP53 interacts with and affects other tumor suppressor activities including CDKN2A (cyclin-dependent kinase inhibitor 2A) [112], BRCA1 [113], and PTEN (phosphatase and tensin homolog [114] (Figure 1). Over-expressing MEG3 induces a significant increase of TP53 protein levels in HCT116 (colorectal cancer) and U2OS (osteosarcoma) cancer cell lines [115]. Further investigation demonstrated that over-expression of MEG3 promotes apoptotic cell death and induces G2/M cell cycle arrest in cervical cancer (HeLa) and retinoblastoma (C33A) derived cell lines [49] through the decrease of CDK1 (cyclin-dependent Kinase 1) and CCNB1 (cyclin B1) levels [49, 63]. Abnormal expression of MEG3 induces apoptosis in HCC derived cell line – PRC/PRF/5 and glioma derived cell lines – U251 and U87 through interaction with TP53 directly by MEG3. MEG3 also affects caspase 3 (CASP3) and p21 (CDKN1A) levels through the activation of TP53 [44, 49]. These findings suggested that the inhibition of tumor cell proliferation by MEG3 is partially due to the induction of G2/M cell cycle arrest and apoptosis by interacting with genes including CCNB1, CDK1 CDKN1A, CASP3 and TP53.

#### MDM2 pathway

MEG3 mitigates TP53 activation and can also be mediated through changing the activity of mouse double minute 2, human homolog (MDM2) (Figure 1). *MDM2* encodes a nuclear E3 ubiquitin ligase that mediates ubiquitination of proteins including tumor suppressor proteins, such as TP53 for degradation [116, 117]. Recently, it has been reported that MDM2 expression is suppressed by MEG3. Inhibition of MDM2 through phosphorylation, acetylation, and sumoylation has an impact on TP53 activity [118–120]. The decreased MEG3 level down-regulates MDM2 expression which leads to an increase of TP53 protein level and enhances TP53 binding to its targeted promoters to stimulate p53-dependent transcriptions [44, 115, 121]. In addition, it has been shown in a mouse model, the increase of MEG3 level up-regulates Tp53 by suppressing Mdm2 [81, 115].

MDM2 itself is also regulated by TP53 at transcription level; therefore, MDM2 and TP53 form an auto-regulatory feedback loop that may be needed to maintain a critical MDM2/TP53 ratio within a cell (Figure 1). Factors that differentially regulate the activities of MDM2 and TP53 may affect cell fate profoundly as they can change the ratio between MDM2 and TP53 [117]. These findings indicated the down-regulation of MDM2 is one of the mechanisms for MEG3 to affect TP53 dependent transcription. Therefore, MEG3 induced apoptosis and anti-proliferative activities in cells may be mediated through the suppression of MDM2 and subsequent activation of TP53 signaling pathway [115, 122].

#### MEG3 and GDF15

MEG3 also enhances a TP53 dependent expression of growth differentiation factor 15 (GDF15), an inhibitor of cell proliferation, which is a member of the transforming growth factor-β (TGFB) superfamily (Figure 1) [123]. Studies reported that GDF15 inhibits proliferation of several cancer cell lines *in vitro* as well as suppresses tumor formation *in vivo* [124, 125]. Zhou et al. demonstrated that GDF15 can be directly affected by MEG3 since re-expression of MEG3 in HCT116 cells induces GDF15 expression level and suppresses cell proliferation [115]. In addition, stimulation of GDF15 expression by MEG3 is through interaction with the GDF15 promoter region [115]. These findings showed that MEG3 not only regulates TP53 but also some TP53 targeted genes such as GDF15.

#### pRb pathway

Retinoblastoma 1 (RB1) protein is an important tumor suppressor which is involved in cancer cell related processes including cell cycle, cell differentiation and apoptosis [126, 127]. RB1 often inhibits tumor cell proliferation by regulating genes required for G to S phase transition and causes G1 cell cycle arrest [128, 129]. Phosphorylation of RB1 by cyclin D (CCND1)/cyclin dependent kinases (CDK4, CDK6) and cyclin E (CCNE1)/CDK2 complexes is necessary to restore the progression of cell cycle [130]. This process is balanced by negative cell cycle regulators, cyclin-dependent kinase inhibitors (CDKNs) which inhibit the G1 phase cyclin–CDK complexes [131]. RB1 can also regulate the stability and the apoptotic function of TP53 via MDM2 [132]. Since MEG3 affects the activities of MDM2 and TP53, it may indirectly affect RB1 mediated tumor suppressing function. In addition, MEG3 may activate RB1 directly by RNA–protein interactions or indirectly by activating CDKN4A, which in turn activates the RB1 to suppress cell proliferation and tumor formation [133].

#### Other MEG3 associated pathways

MEG3 has also been found to suppress cell proliferation and promote apoptosis through the VEGF pathway, Wnt/β-catenin pathway and TGF-β pathway in some cancers. For example, Gordon et al. observed a significantly increased microvessel formation in the brain together with the elevated expression of genes involved in VEGF angiogenic pathway in a MEG3 KO mice brain compared with normal wild type [81]. The increase of VEGF pathway activity in MEG3 KO mice brain suggests that MEG3 may play an important role in the progression for tumors like meningioma. In addition, one recent study showed that overexpression of MEG3 suppresses cell proliferation and promotes apoptosis by reducing the Wnt/β-catenin pathway activity in retinoblastoma cell lines. Treating the cells with a Wnt/β-catenin pathway activator reverses MEG3 over-expression induced anti-proliferation activity in retinoblastoma cell lines [77]. Using a modified chromatin oligo affinity precipitation method, Mondal et al. found some of the genes involved in TGF-β pathway interact directly with MEG3 [134]. It has been suggested that MEG3 regulates the expression of its target genes through the formation of RNA–DNA triplex structures [134], which may be a general mechanism for gene regulation mediated by lncRNAs.

#### MEG3 and drug resistance in cancers

Many studies have shown that processes related to drug resistance are modulated by lncRNAs [17]. For example, Yang et al. showed a significant decrease of MEG3 level in cisplatin-resistant A549/DDP lung cancer cells [18]. Furthermore, inducing the expression of MEG3 was able to re-sensitize the A549/DDP cells to cisplatin *in vitro*. The same study also demonstrated MEG3-mediated chemosensitivity was associated with the induction of cell cycle arrest and increased apoptosis through genes in the WNT/βcatenin signaling pathway such as TP53, β-catenin, and survival [18, 77]. Similar to what has been observed *in vitro*, a decrease of MEG3 level has been found in cisplatin-resistant human lung adenocarcinoma (LAD) tissues accompanied with decreased TP53 and increased Bcl-xl protein levels [38]. Therefore, the level of MEG3 could be used as a potential biomarker to gauge the response to cisplatin based chemotherapy in lung cancer.

#### Future prospect of MEG3 in human cancer

Based on findings described in literatures, MEG3 is an RNA-based tumor suppressor and is involved in the etiology, progression, and chemosensitivity of cancers [2, 19, 20, 28, 106, 135–137]. Its activity is mediated through both TP53-dependent and TP53-independent processes (Figure 1). MEG3 interacts with a number of well characterized tumor-related genes including TP53, MDM2, GDF15, RB1 and TGFB. Differential expression of MEG3 between normal and different grades of cancers offers the possibility of using MEG3 to assess the stage and prognosis of cancer [2, 138, 139]. The spectrum of lncRNAs including MEG3 in tissue samples can be measured by various profiling methods including microarray, next generation sequencing, and qPCR [140–142]. This may lead to a noninvasive, inexpensive and reliable method to monitor cancer progression and assess the prognosis of disease [138, 139, 143].

Modulating the levels of lncRNAs such as MEG3 through methods including over-expression, RNAi mediated gene silencing, or by small molecule inhibitors for cancer therapy looks promising in *in vitro*. Even though attempts have been made to improve the delivery system; to use lncRNA as a therapeutic target *in vivo* is still challenging, especially in reducing the off-targets effect and delivering the expressing vector or RNAi into specific cells [136, 138, 139, 144].

In summary, some lncRNAs like MEG3 are strongly associated with the clinicopathological outcome of various cancers. Lost or decreased expression of MEG3 is common in human cancers. The effects of MEG3 expression on cancer development are well-documented and MEG3 has been attributed as a tumor suppressor based on its involvement in tumor development. MEG3 can be used as a promising target for cancer diagnosis, prognosis and treatment; however, more fundamental work such as efforts to reduce immune response, minimize off-targets effects, and develop more effective targeted deliver system is needed in order to further develop possible clinical applications.

## SUPPLEMENTARY MATERIALS TABLE



## Abbreviations

AML: Acute myeloid leukemia
CASP3: Caspase 3
CCND1: Cyclin D
CCNB1: Cyclin B1
CDK1: Cyclin-dependent Kinase 1
CRC: Colorectal cancer
DFS: Disease free survival
DMR: Differentially methylated region
ESCC: Esophageal squamous cell carcinoma
GC: Gastric cancer
GDF15: Growth differentiation factor 15
HCC: Hepatocellular carcinoma
IG-DMR: Intergenic differentially methylated region
KO: Knockout
LAD: Lung adenocarcinoma
LncRNA: Long noncoding RNA
MDM2: Mouse double minute 2, human homolog
MEG3: Maternally expressed gene 3
miRNA: microRNA
NFPA: Nonfunctioning pituitary adenoma
NSCLC: Non-small cell lung cancer
OS: Overall survival
RB1: Retinoblastoma 1
snoRNA: Small nucleolar RNA
TNM: Tumor node metastasis
