# Supplementary material for: Potential applications of MEG3 in cancer diagnosis and prognosis
Source: Oncotarget. 2017 Aug 4;8(42):73282–95. doi: 10.18632/oncotarget.19931 (PMC5641212; doi:10.18632/oncotarget.19931)
Supplement: Supplementary file 1 [file oncotarget-08-73282-s001.pdf]

Potential applications of MEG3 in cancer diagnosis and prognosis

SUPPLEMENTARY MATERIALS

Supplementary Table 1: Changes of MEG3 expression level in different human cancer tissues. See Supplementary_Table_1
